# Asthma and COPD in primary health care, quality according to national guidelines: a cross-sectional and a retrospective study

**DOI:** 10.1186/1471-2296-9-36

**Published:** 2008-06-19

**Authors:** Siw Carlfjord, Malou Lindberg

**Affiliations:** 1 Linköping University, Department of Medicine and Health Sciences, SE-581 83 Linköping, Sweden; 2 County Council of Östergötland, R&D Department of Local Health Care, SE-581 85 Linköping, Sweden

## Abstract

**Background:**

In recent decades international and national guidelines have been formulated to ensure that patients suffering from specific diseases receive evidence-based care. In 2004 the National Swedish Board of Health and Welfare (SoS) published guidelines concerning the management of patients with asthma and COPD. The guidelines identify quality indicators that should be fulfilled. The aim of this study was to survey structure and process indicators, according to the asthma and COPD guidelines, in primary health care, and to identify correlations between structure and process quality results.

**Methods:**

A cross-sectional study of existing structure by using a questionnaire, and a retrospective study of process quality based on a review of measures documented in asthma and COPD medical records. All 42 primary health care centres in the county council of Östergötland, Sweden, were included.

**Results:**

All centres showed high quality regarding structure, although there was a large difference in time reserved for Asthma and COPD Nurse Practice (ACNP). The difference in reserved time was reflected in process quality results. The time needed to reach the highest levels of spirometry and current smoking habit documentation was between 1 and 1 1/2 hours per week per 1000 patients registered at the centre. Less time resulted in fewer patients examined with spirometry, and fewer medical records with smoking habits documented. More time did not result in higher levels, but in more frequent contact with each patient. In the COPD group more time resulted in higher levels of pulse oximetry and weight registration.

**Conclusion:**

To provide asthma and COPD patients with high process quality in primary care according to national Swedish guidelines, at least one hour per week per 1000 patients registered at the primary health care centre should be reserved for ACNP.

## Background

In recent decades international and national guidelines have been formulated to ensure that patients suffering from specific diseases receive evidence-based care [1-4]. In 2004 the National Swedish Board of Health and Welfare (SoS) published guidelines concerning the management of patients with asthma and COPD [5] based on Donabedian's theory regarding evaluation of the quality of medical care, which focuses on aspects of structure, process and outcome [6]. The guidelines describe structure indicators as basic conditions for providing good care, process indicators as measuring what is actually performed, and outcome indicators as measuring the effects on health and well-being. All of them represent quality indicators [5]. To ensure care of equally high quality for all patients throughout the country, the guidelines suggest that in the management of asthma and COPD in primary health care, quality indicators for all three aspects should be fulfilled [5]. Measuring lung function and documentation of smoking habits are important process indicators. Measuring lung function using spirometry [7] is important both for diagnosis of the diseases and for providing optimal pharmacological treatment [1,2], and the presence of nurses with specialised education and training ensures more frequent use of spirometry [8]. Regarding smoking, there is a strong relation between the development of COPD and smoking habits, and the most effective action is smoking cessation [9,10]. Giving up smoking is also of considerable benefit to asthma patients [11].

Many primary health care centres have developed Asthma and COPD Nurse Practices (ACNPs) [5,12,13] to provide good management for patients suffering from asthma or COPD. This organisation of nurse practices in primary health care is considered to be cost effective, since examination of lung function and patient follow-up are done mainly by nurses [14,15]. Specially trained nurses providing care to patients with specific diagnoses has become common [16-18]. A study from the UK showed that primary care nurses spend a mean of 6.6% of their time caring for patients with respiratory diseases, and 68% of this time is devoted patients with chronic respiratory illness [19]. The Swedish Association for General Practitioners (SFAM) recommends allocation of at least 30 minutes per week per 1000 patients registered at the health care centre for a satisfactory Asthma Nurse Practice (ANP) [20,21].

The aim of this study was to survey structure and process indicators, according to the asthma and COPD guidelines, in primary health care, and to identify correlations between structure and process quality results.

## Methods

The study was performed in the county of Östergötland with about 420 000 inhabitants. The aspects of structure and process quality recommended in the national Swedish guidelines [5], and focused on in the study, are illustrated in table 1.

**Table 1:** Overview of indicators recommended in guidelines and investigated in the study.

Indicators	Recommended structure indicators for asthma/COPD	Recommended process indicators for asthma	Recommended process indicators for COPD	Investigated in asthma records	Investigated in COPD records
ACNP	X			X	X
Spirometer	X			X	X
Nebulizer	X			X	X
Pulse oxymeter	X			X	X
Time	X			X	X
Nurse education level	X			X	X
Visits per time period per patient		X	X	X	X
Proportion of patients who had performed PEF test		X		X	
Proportion of patients who had performed spirometry		X	X	X	X
Proportion of patients who had performed spirometry with Reversibility test			X		X
Proportion of records containing smoking documentation		X	X	X	X
Proportion of patients with registered weight			X		X

### Structure quality – cross-sectional

A computer-based survey instrument, Publech^® ^Survey 5.6, was used to design a questionnaire that was e-mailed to all 42 primary health care centres in Östergötland, Sweden, in January 2006. The questions were based on quality indicators stipulated in the national guidelines, see table 1 (fixed-response questions and open-ended questions). The manager at each health care centre was asked to answer the questionnaire.

### Process quality – retrospective

The process quality was measured by retrospectively examining medical records. All centres used structured computer-based medical records with similar search terms. The study sample comprised a random sample of medical records of patients suffering from asthma or COPD who had had contact with a GP in primary health care during 2004 or 2005. Only records from individuals born in 1985 or earlier were included. One of the 42 centres was excluded for organisational reasons. A power calculation was performed to estimate the number of medical records needed to obtain statistically significant differences of 20 per cent (significance level p ≤ 0.05, power 80 per cent) between the groups according to time reserved for ACNP, and each process indicator. The calculation indicated that 1000 medical records, representing 0.24 per cent of the total number of patients registered in primary health care in the county council in 2006, would be sufficient. Thus the number of forms for examination of medical records sent to each centre was 0.24 per cent of the number of patients registered at the centre, in total 1052 forms, half for asthma records and half for COPD records. From computer generated lists of asthma patients and of COPD patients according to age, the requested number of patients was obtained in a randomised fashion. The questions on the forms concerned the process indicators listed in table 1. All questions had a fixed-response format.

The forms for examination of medical records were sent to the nurse working at the ACNP at each centre, and this nurse was asked to perform the examination. Only centres that answered the structure survey questionnaire and also completed the medical records examination assessing process results were included in the results of the study.

### Analysis

Statistical analyses were done using the computer-based analysis program SPSS version 14.0. Differences between means were analysed using the independent samples t-test. The degree of linear association between measures was assessed using Pearson's correlation coefficient. Statistical significance was defined as a p-value ≤ 0.05.

### Ethical aspects

According to Swedish law, The Act concerning the Ethical Review of Research Involving Humans (SFS 2003:460) from the Ministry of Education an Cultural Affairs, the present study requires no ethical approval.

## Results

Thirty-nine (93 per cent) of the 42 centres included answered the structure questionnaire and returned the forms used for examining the medical records. The number of patients at the different health care centres varied from 3785 to 20763, with an average of 10000.

### Structure quality results

All 39 centres reported having an ACNP with a GP in charge, and access to a spirometer and a pulse oximeter. Two centres lacked a nebulizer. Nurses with education and training at the university level in the area of asthma and COPD were found in 26 of the 39 centres.

**Figure 1:**
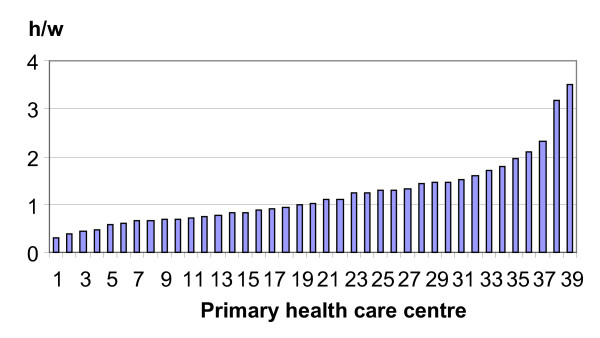
Time (hours per week per 1000 registered patients) reserved for ACNP at each primary health care centre.

The amount of time reserved for ACNP in the 39 centres is presented in figure 1.

### Process quality results

In total, 997 of 1052 forms were completed and returned (95 per cent), of which 497 were asthma forms and 500 were COPD forms.

The results showed that examination of lung function with PEF or spirometry, as well as documentation of smoking habits, were more frequently carried out in the ACNP than at GP consultations (table 2).

**Table 2:** Visits to GPs and ACNPs: process quality indicators documented in medical records according to diagnosis.

Indicators	Asthma GP visits	Asthma ACNP visits	COPD GP visits	COPD ACNP visits
	n = 311	n = 236	n = 376	n = 260
	%	%	%	%
Spirometry	5	98	8	97
PEF*	56	90	-	-
Smoking habits documentation	28	78	55	85

*Peak Expiratory Flow data were searched for only in medical records of patients with an asthma diagnosis

### Correlations between structure and process

When comparing time reserved for ACNP with the percentage of patients in both the asthma group and the COPD group for whom the different process indicators were fulfilled, there was an increase in several of the indicators when the amount of time reached one hour per week per 1000 patients (table 3). With longer time, > 1 1/2 hours per week, no further improvement was found in the asthma group. A lower proportion of medical records contained documentation of current smoking habits at the centres with > 1 1/2 hours per week compared to those reserving between 1 and 1 1/2 hours.

**Table 3:** Time reserved for ACNP, in hours per week per 1000 registered patients, compared with process quality results documented in medical records according to diagnosis. Number of centres in parentheses.

Indicators	Asthma		Asthma		Asthma	COPD		COPD		COPD
	<1 h/w (19)		1–1 1/2 h/w (11)		>1 1/2 h/w (9)	<1 h/w (19)		1–1 1/2 h/w (11)		>1 1/2 h/w (9)
	n = 253		n = 142		n = 102	n = 256		n = 142		n = 102
	%		%		%	%		%		%
Spirometry	36	p = 0.000	68	p = 0.01	52	44	p = 0.003	60	p = 0.54	64
Smoking habit documentation	39	p = 0.000	57	p = 0.09	46	59	p = 0.02	70	p = 0.12	61
PEF^1^	53	p = 0.000	74	p = 0.22	67	-		-		-
Reversibility test^2^	-		-		-	22	p = 0.19	28	p = 0.42	24
Pulse oximetry^2^	-		-		-	22	p = 0.35	20	p = 0.045	33
Weight^2^	-		-		-	14	p = 0.554	18	p = 0.016	28

1 = only searched for in medical records of patients with an asthma diagnosis

2 = only searched for in medical records of patients with a COPD diagnosis

There was no correlation between nurses' level of education and fulfilment of the process indicators, or between number of patients registered at each centre and frequency of examination of lung function, documentation of smoking habits, or registration of weight and pulse oxymetry.

The number of contacts each patient had had with the ACNP showed a linear association with time reserved for ACNP at each centre (p < 0.001).

## Discussion

This study was a survey of the management of patients with asthma and COPD in primary health care. The results show that structure quality in terms of time reserved for ACNP is associated with the process quality of care as described in the Swedish national guidelines [5]. Measuring quality according to national guidelines is a strategy for encouraging improvement in health care. In Sweden, high quality does not generate economic benefits for the centre, but it stimulates 'intrinsic motivation', described as a desire to perform as well as possible and a sense of belonging to a group that behaves according to a shared set of values [22].

Previous studies reported that patients who consulted nurse practitioners received longer consultations, were given more information, and were generally more satisfied compared with those who had GP consultations [23,24]. The results of this study show that examination with spirometry is mainly performed in ACNP, and is infrequently done by GPs. In Spain, spirometry has been found to be underused in primary care, which affects the quality of COPD care [25,26]. Documentation of current smoking habits, which indicates that this subject has been discussed with the patient, is more frequent in ACNP records than in GP records. It is important to ask patients about their smoking habits, and to try to convince them of the benefits of smoking cessation, since it has been shown that the simple act of asking about smoking habits leads to smoking cessation in a number of patients [27]. There is reason to believe that the amount of time reserved for ACNP is an important factor in providing good quality management of these groups of patients. Another way to improve quality could be through the use of primary care models as described by Meulepas et al [28].

The optimal time for maintaining good quality regarding spirometry and current smoking documentation seems to be between 1 and 1 1/2 hours per week. More than 1 1/2 hours per week resulted in larger proportions of COPD patients being examined with pulse oximetry and having their weight registered, but not in higher levels of spirometry, or more frequent documentation of smoking habits in either of the groups. Increased time resulted in more frequent visits to the ACNP by each patient. This could be considered higher quality for those particular individuals, and it is also possible that these centres manage patients with more severe forms of the diseases and therefore allocate more time to each patient. The estimation of time spent for ACNP was made by the manager at each centre, based on the economic resources allocated, and thus is considered a true value.

A strength of this study is the high response level and that almost all primary health care centres in the county council participated. A weakness is the large number of different individuals who examined the medical records, and that in many centres the nurses themselves reported their findings. However, the medical records are structured, and we believe it unlikely that the nurses reported false findings. Assessment of structure quality was carried out as a cross-sectional study, while process quality was studied retrospectively during a two-year period. This means that structure quality factors might have changed during the two-year period.

The Swedish guidelines [5] do not provide any benchmark concerning the structure or process indicators, which is why it is not possible to determine if the levels obtained are sufficient to provide optimal care. Aspects of outcome in terms of patient satisfaction or well-being were not considered in the present study, since this type of data cannot be found in the medical records. Comparing outcome with structure and process indicators would require a more extensive survey. One such study performed in the Netherlands showed that combining different disciplines in one model improved the care process and patient outcomes regarding COPD patients [29].

The most important finding of this study is the determination of the amount of time needed for ACNP in order to obtain the best process quality outcomes. We suggest that each primary health care centre should reserve at least one hour per week per 1000 registered patients in order to offer optimal management.

To our knowledge, this study is the first attempt to determine the level of time needed for ACNP in primary health care. Further research is needed to confirm the results of this study, as well as to investigate the outcomes for quality results in terms of effects on health and well-being.

## Conclusion

The amount of time reserved for ACNP seems to be an important factor in providing good quality management of asthma and COPD patients. To provide these groups of patients with high process quality in primary care according to national Swedish guidelines, at least one hour per week per 1000 patients registered at the primary health care centre should be reserved for ACNP.

## Competing interests

The authors declare that they have no competing interests.

## Authors' contributions

SC contributed to the conception and design of the study, to acquisition, analysis and interpretation of data, and also to drafting the manuscript. ML contributed to the conception and design of the study, to analysis and interpretation of data, and to critical revision of the manuscript.

## Pre-publication history

The pre-publication history for this paper can be accessed here:


